# Evaluating the methods used to examine sitting breaks and their influence on mental load, physical strain, and cognitive performance - a scoping review

**DOI:** 10.3389/fphys.2026.1755356

**Published:** 2026-02-18

**Authors:** Marion Freyer, Charline Jost, Sylvia Jankowiak, Kim-Aljoscha Bressem, Janice Hegewald

**Affiliations:** Federal Institute for Occupational Safety and Health (BAuA), Berlin, Germany

**Keywords:** active breaks, cognition, EEG, EMG, muscle activity, prolonged sitting, sedentary behaviour

## Abstract

**Background:**

Long periods of sitting characterize modern working life and are associated with increased health risks. Integrating short activity breaks may counteract these effects. This scoping review examines the effects of brief bouts of physical activities on cognitive performance and neurophysiological parameters.

**Methods:**

A comprehensive search in PubMed and EBSCOhost identified experimental and field studies with adult participants examining the effects of interrupting sitting periods with short physical activities on cognitive performance, neurophysiological parameters (EEG), and muscle activity (EMG). Studies focusing solely on standing or posture changes were excluded. Study quality and internal validity were assessed using the revised Cochrane tool for assessing the risk of bias in randomized trials. A narrative synthesis summarised the findings.

**Results:**

Eighteen studies met the inclusion criteria, with a total of 694 participants aged between 20 and 50 years. Of these, only one study examined the influence on EEG and found that walking breaks increased attention-related brain activity, as indicated by a higher P3 amplitude. However, behavioural performance remained unchanged. Two studies examined muscular parameters using EMG, one of which observed a reduction in fatigue. Cognitive performance was assessed in 16 studies. Only two studies used standardized and realistic work tasks to keep participants engaged during the sitting periods. The results varied widely and only occasionally showed a positive influence of movement breaks on cognitive function.

**Conclusion:**

Reasons for the heterogeneity of the results on cognitive performance may lie in different study designs, types of intervention, and outcome measurements. Another factor is the tasks assigned during the sitting phases. Variations in mental load during the different tasks cannot be ruled out, which in turn may influence cognitive performance outcomes after the interventions. The limited number of studies, which often had small sample sizes, and the considerable methodological heterogeneity do not allow for definitive conclusions. Nevertheless, the review provides some evidence that interrupting prolonged sitting with short breaks of physical activity may help maintain cognitive performance and muscle health. These findings underscore the need for more rigorous, ecologically valid research to better understand the health effects of interrupting sedentary activities.

**Systematic Review Registration:**

https://www.crd.york.ac.uk/PROSPERO/view/CRD42025638431, identifier CRD42025638431.

## 1 Introduction

In today’s increasingly digital and office-based workplaces, prolonged sitting has become an all-too-common and frequently overlooked aspect of modern working life. The results of a representative survey of employed people in Germany have shown that 16% of participants sit between 25 and 35 h per week during work and 19% even more than 35 h (Schmidt et al., 2025). It should be noted that these times were recorded on the basis of self-reporting. Thus, these results may be underestimated (Prince et al., 2020). According to a global meta-analysis of device-measured sedentary behaviour, employees spend approximately 60% of their working hours sitting—an even greater proportion is observed among office workers (Prince et al., 2019). Even longer sitting times were observed when working from home (Chaudhary et al., 2024; Polspoel et al., 2025; Sauter et al., 2025; Schöne et al., 2025).

Prolonged sedentary time is generally associated with an increased risk for various diseases like type 2 diabetes and obesity as well as all-cause and cardiovascular mortality, even after accounting for physical activity (PA) levels (Proper et al., 2011; Wilmot et al., 2012; Barlow et al., 2016; Patterson et al., 2018). The long-term consequences of sedentary behaviour (SB) may include neurodegenerative diseases, which result from structural changes in certain regions of the brain and the deterioration of associated cognitive functions (Chandrasekaran et al., 2021; Kardys et al., 2022). SB during work also appears to influence musculoskeletal complaints. A higher prevalence of neck and shoulder muscle complaints is observed among individuals who report sitting for 25 to less than 35 h per week at work and who do not engage in any PA during their leisure time (Dang et al., 2024). As sitting time increases, physical discomfort, especially in the lower back, increases. So does the number of small and large postural shifts on the office chair in an attempt to compensate for this discomfort (Waongenngarm et al., 2020).

To offer employees flexible working options and break up long periods of sitting, many offices introduced sit-stand desks. These lead to behavioural changes, such as reductions in sitting time and increases in standing time, as well as a decrease in discomfort. However, the benefits for cardiometabolic health are only minor (Chambers et al., 2019). Therefore, standing while working may not be sufficient to promote health. In this context, the WHO emphasises the importance of reducing sedentary time and replacing it with any level and any length of PA (World Health Organization, 2020). Even short periods of activity can have a positive effect on health; every bit of movement counts.

The impact of sedentary time may be moderated by cardiorespiratory fitness and overall PA (Barlow et al., 2016). Engaging in regular PA—regardless of intensity—helps counteract the effects of sedentary time, lowering the risk of premature death and improving cardiometabolic health (Chastin et al., 2019; Ekelund et al., 2019; Ekelund et al., 2020). Staying active and reducing SB also supports cardiovascular function, boosts muscle strength and immunity, and reduces the risk of chronic diseases such as diabetes, heart disease, and certain cancers (Reiner et al., 2013; Miller et al., 2016; Brakenridge et al., 2024). Several studies have explored the relationship between movement and cognitive performance (CP). A meta-analysis found a generally small positive effect of exercise on cognitive function (Chang et al., 2012). Immediately after PA, cognitive benefits were observed only with light to moderate intensity exercises. The effects were more pronounced for tasks involving attention and executive functions. In terms of fitness level, positive effects were observed in both low-fit and high-fit individuals, while moderately fit individuals showed no effect on cognition. When tested after a delay, light-intensity exercise had a negative, whereas moderate-to high-intensity exercise a positive effect. Compared to an active control group, physical strength training over a 12-week period improved tasks related to working memory (WM) and recall (Kardys et al., 2022). In terms of neurocognitive components measured with electroencephalography (EEG), such as processing speed and attentional capacity (P3 latency and amplitude), it has been shown that exercise can slightly increase P3 amplitude and reduce latency (Kao et al., 2022b). This indicates improved cognitive processing. These effects vary depending on age, intensity and duration of the exercise: older adults benefit more, while high-intensity interval training (HIIT) is less effective than aerobic or combined exercises. Additionally, shorter training sessions (up to 20 min) improve processing speed more effectively than longer sessions, while longer sessions (>20 min) improve attentional capacity more effectively (Kao et al., 2022b).

There are various physiological and neurocognitive mechanisms explaining why physical activity following periods of sitting may enhance cognitive performance. For example, acute aerobic exercise increases cerebral blood flow and triggers the release of catecholamines like dopamine and noradrenaline, supporting neural efficiency and executive functions (Sudo et al., 2022). However, during high-intensity exercise, the oxygen supply to the brain decreases, which in itself does not necessarily lead to impaired cognitive performance. Nevertheless, cognitive performance can be affected by how quickly the oxygen supply to the brain recovers after exercise (Sudo et al., 2022). On a neurobiological level, physical activity promotes neuroplasticity through increased expression of neurotrophic factors such as brain-derived neurotrophic factor (BDNF), supporting learning and memory (Sousa Fernandes et al., 2020). Together, these mechanisms provide a plausible explanation for why even short bouts of movement or interruptions of prolonged sitting may lead to measurable improvements in cognitive performance.

In modern work environments, where prolonged sitting is common, strategies to mitigate the negative effects have gained increasing attention. One such approach involves sitting interruptions with PA, which may influence cognitive and physiological parameters. However, findings on the impact of interrupting work-related prolonged sitting are inconsistent, which may be due to their heterogeneous designs. In a highly demanding workplace, it was found that different break types, such as boxing, biking or relaxation, had no effect on mood or attention but on decision making compared to usual or no breaks (Wollseiffen et al., 2016; Scholz et al., 2018). Nevertheless, the resting EEG after the break intervention and the cognitive measurements showed increased relaxation after boxing and biking compared to other breaks. Although prolonged sitting at work is likely to cause fatigue, which can be reduced by taking short walking breaks, no improvement in CP was found (Wennberg et al., 2016). The situation is different when it comes to physical discomfort and complaints. Workplaces that are actively designed to combine sitting, standing and walking or implementing breaks with stretching exercises have a mitigating effect on muscular discomfort (Nakphet et al., 2014; Osama et al., 2015; Kar and Hedge, 2020). Further research into the short- and long-term effects of postural shifts and active breaks at work revealed a reduction in recovery time and recurrence, as well as a decrease in musculoskeletal pain (MSP) after 3 months (Akkarakittichoke et al., 2021; Dzakpasu et al., 2023). In contrast, increasing standing time increased MSP (Dzakpasu et al., 2023), though this effect was no longer visible after 12 months. Complementary findings from studies utilizing infrared thermography also highlight the advantages of active breaks in alleviating muscular strain during extended sitting periods (Sortino et al., 2024). These findings highlight the fundamental value of incorporating regular work breaks.

Despite these findings, the effects of easily implementable, health-promoting interventions on CP and physical health in the workplace remain unclear. Many of the interventions examined are either too complex to integrate into everyday working life and require specialized equipment, extensive supervision, very high physical exertion, or substantial time resources. Also, the outcomes/measures examined often rely on subjective self-reports rather than standardized, objective indicators of physiological or cognitive strain. As a result, the practical applicability and comparability of findings across studies remain limited. A meta-analysis concluded that the influence of interrupting long sitting periods with short movement breaks on cognitive performance remains inconclusive (Li et al., 2022). However, one criticism of the meta-analysis is that the cognitive areas of executive function, memory, and attention were not analysed separately, but together. Despite investigating the effects of PA, many studies do not incorporate a preceding sedentary period, which is essential for accurately assessing the impact of movement interruptions. The lack of methodological consistency and interdisciplinary synthesis underscores the importance of a systematic review to consolidate current findings and identify patterns across different approaches.

This scoping review aims to systematically explore how interrupting prolonged sitting with short bouts of PA may affect CP and muscular strain in healthy working-age adults. Additionally, the review should highlight any methodological limitations of the existing research to inform and improve the design of future experiments. Guided by the PECOS framework (Morgan et al., 2018), we focus on laboratory and field studies that compare movement-based sitting breaks - such as walking, stepping, cycling, or resistance exercises - performed after a defined period of sitting, against a prolonged-sitting control condition. Unlike existing reviews that focus on behavioural aspects and their underlying physiological mechanisms (Chandrasekaran et al., 2021; Chueh et al., 2022; Li et al., 2022), this review integrates psychophysiological outcomes (e.g., mental load via EEG), behavioural performance (reaction time and accuracy), and muscular activity via electromyography (EMG). In particular, this review seeks to uncover systematic differences by analysing variations in intervention type, duration and frequency. It also aims to identify the best available methods for objectively examining the psychophysiological impact of sitting interventions.

## 2 Materials and methods

### 2.1 Registration

The record of this scoping review has been registered in January 2025 in the international prospective register of systematic revies (PROSPERO) with the registration number CRD42025638431. At the start of the title abstract screening in March 2025, we decided to specify the exclusion criteria with regard to studies without prolonged sitting prior to exposure. This change was registered with PROSPERO in April 2025.

### 2.2 Inclusion and exclusion criteria

The PECOS framework was used as a guide for screening potentially relevant articles (Morgan et al., 2018).

Population: Studies involving healthy adults of working age (18–67 years) were included. Studies involving participants with known health issues (e.g., pre-existing mental illness, obesity, physical limitations), adolescents and children, individuals over 67 years of age, or animal studies were excluded.

Exposure: We included studies that examined the effects of sitting breaks after periods of sitting with different types of movement on psychophysiological, behavioural, and muscular parameters. The movements could include walking/running, stepping, cycling, body resistance exercises, or similar activities performed directly at the workplace/desk or away from the workplace. We included studies that collected data for the outcomes after the movement break. Studies focusing exclusively on effects during movement, as well as those examining only transitions from sitting to standing or posture changes were excluded.

Control: Studies describing prolonged sitting as comparator were included. We excluded studies without a comparison group those lacking an explicitly reported sitting phase prior to the activity, while not specifying any minimum sitting duration. This applied, for example, to studies that examined the pure effect of exercises in comparison with no exercises. We made no restriction regarding the minimum sitting time.

Outcomes: Studies that reported results for the following parameters were included: 1) EEG (time- or frequency-based), 2) cognitive behavioural parameters (reaction times and error rates) in cognitive tests, and 3) recording of muscle activity, tension, or fatigue.

Study design: The search included both randomized and non-randomized laboratory studies (crossover- or parallel-group-design) with controlled movement protocols, as well as field studies with natural interruptions of sitting that include everyday work movements. The latter refers, for example, to errands in the office building or participation in active breaks offered by the employer. Only peer-reviewed, scientifically published articles were eligible for inclusion; dissertations, theses, and other forms of grey literature were not considered ensuring methodological rigor, transparency, and reproducibility.

In summary, we defined eight categories of exclusion criteria: wrong population, wrong publication type, wrong exposure, wrong comparison, wrong outcome, wrong study design, foreign language, and others.

### 2.3 Search strategy and process

To answer the research question, a comprehensive search was conducted on 30 January 2025, using PubMed (including MEDLINE, OLDMEDLINE and PubMed Central) and EBSCOhost (including PsycInfo and PsycArticles). No time or language restrictions were applied at this stage of search. During the screening process, studies not published in English or German were excluded.

When developing the search string, we focused on terms related to sedentary work, interruptions to sitting, movement, cognitive performance, psychophysiological parameters, and muscular strain in accordance with our defined PECOS scheme. By successively adding relevant terms or removing terms that did not provide any quantitative additional benefit, we developed the following search string:

(seated OR sedentar* OR sitt*) AND (electroence* OR electromyo* OR cognit* OR executive OR “information process*” OR “Neuropsychological Tests” [Mesh] OR Cognition [Mesh] OR “Reaction Time/physiology” [Mesh] OR “Attention” [Mesh] OR “Inhibition, Psychological” [Mesh] OR “Electroencephalography” [Mesh]) AND (step* OR walk* OR treadm* OR break* OR interrupt* OR “physic* exercis*” OR “physic* activ*”).

For both databases the search string was identical; only the identification for mesh terms was adjusted for each of them (‘ [Mesh]’ for PubMed and ‘MM’ for EBSCOhost). This search strategy was validated by identifying studies that we had predefined as relevant (Chrismas et al., 2019; Chandran et al., 2023).

The titles and abstracts of the studies found using the search strategy were imported into Rayaan (Ouzzani et al., 2016) and automatically suggested duplicates were manually checked and removed. In a first step, MF and CJ initially screened titles and abstracts of the first 40 studies independently and blinded to identify those that could meet the above inclusion criteria (coded as ‘yes’, ‘no’, or ‘maybe’). A quality check of the preselection was carried out to ensure consistency between these two reviewers. This clarified whether all inclusion and exclusion criteria were sufficiently clear for the further screening. At this point, we decided to clarify the exclusion criteria regarding studies without prolonged sitting prior to exposure. In summary, MF screened all title abstracts and CJ screened 20% of them independently. SJ was consulted if no consensus had been reached in all screening stages. All full texts were double-screened blinded for quality control, with MF checking all studies and CJ, SJ and KB dividing the same studies among themselves, each reviewing 33%. One main reason for exclusion was noted. In case of uncertainty, the inclusion of questionable studies was discussed within the project team. The search was supplemented by a manual screening of thematically related reviews found during the search. In addition, we systematically screened the reference lists of all included studies.

### 2.4 Data extraction

A standardized, pre-tested form was used to extract the relevant data from the included studies for synthesis of the evidence. The summary information and overall results of all included studies were extracted by MF and supplemented by CJ and KB. The information extracted includes: publication information (first author, year of publication, country), study design, study population (sample size and demographic data of participants), measured cognitive domain, details of experimental procedure and exposure (type and intensity, duration and frequency). No explicit or apparent missing data were reported in the included studies.

### 2.5 Risk of bias assessment

Internal validity and quality were assessed using the RoB two tool (Revised Cochrane Risk-of-Bias Tool for Randomised Trials) (Sterne et al., 2019). Bias was assessed for each outcome relevant for the research question of this review in five different domains (*randomisation process, deviations from the intended interventions, missing outcome data, measurement of outcomes, selection of reported results*) each with at least one signalling question. These assessments resulted in ratings of ‘low risk of bias’, ‘some concerns’ or ‘high risk of bias’ within the respective domain, and subsequently to an overall risk of bias assessment. This was based on algorithms using responses to specific signalling questions. As both crossover and parallel-group designs were identified, the respective versions of the RoB two tool were applied separately to each included study. MF and CJ independently assessed the studies for bias. Any disagreements were resolved by SJ.

### 2.6 Qualitative synthesis

A narrative synthesis summarised the results of the included studies by describing the study population, the exposure, the measurement of the outcomes, and the influence of exposure on the outcomes compared to the control condition. The focus was on the differences in psychophysiological, behavioural, and muscular outcomes, as well as the differences depending on the type, duration, and frequency of sitting breaks.

## 3 Results

### 3.1 Study selection

**FIGURE 1:**
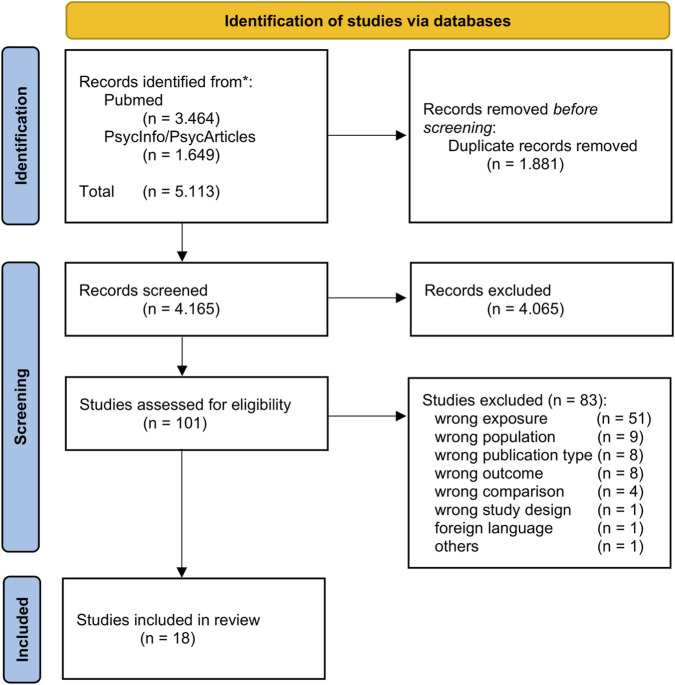
PRISMA 2020 flow diagram of the search process.

The database search yielded 5.113 records. After removing duplicates, 4.165 records remained for title and abstract screening. Of these, 100 articles were assessed in full text for eligibility, and 18 studies met the inclusion criteria. In addition, we screened the reference lists of all the included studies to identify any further relevant publications. Only one relevant article was found in this process, and it was subsequently subjected to full-text screening. However, it was excluded based on our predefined exclusion criteria. The most common reason for excluding the 83 remaining studies was ‘wrong exposure’ (62%), as these studies only examined the effect of exercise versus sitting on various outcomes without taking into account periods of sitting prior to an exercise break. The exclusion reason ‘others’ refers to a study that was withdrawn by the authors and published elsewhere (Thirunavukarasu et al., 2024). This newly published study was included in our review (Thirunavukkarasu et al., 2025). Manual screening of thematically relevant reviews from the search results did not yield any additional relevant studies. Figure 1 shows a PRISMA flow diagram of the search process. Supplementary Table S1 in the Supplementary Material provides an overview of the excluded studies.

### 3.2 Study characteristics

We included 18 studies, 13 of which had a randomised crossover design (Bergouignan et al., 2016; Sperlich et al., 2018; Vincent et al., 2018; Chrismas et al., 2019; Charlett et al., 2021; Heiland et al., 2021; Chandran et al., 2023; Horiuchi et al., 2023; Wu et al., 2023; Kuo et al., 2024; Chueh et al., 2025; Thirunavukkarasu et al., 2025; Yu et al., 2025), four had a randomised parallel group design (McLean et al., 2001; Ding et al., 2020; Niedermeier et al., 2020; Yu et al., 2022) and one had a within-person encouragement approach (Giurgiu et al., 2024). The main characteristics of the included studies are presented in Table 1.

**TABLE 1:** Study characteristics.

First author (year), country	Study design	Sample size	Female %	Mean age, (SD)	Cognitive domain	Physical activity	Intensity	Duration experiment (minutes per day)	Duration exposure (minutes after sitting)
Outcome: cognitive performance									
Bergouignan et al. (2016)*, USA	Crossover	30	70	30.0 (5.6)	Executive function, attention	Walking	Moderate	420′	6*5′ every 60′ or 1*30′ before sitting
Chandran et al. (2023), India	Crossover	21	19	26.7 (2.6)	Executive function	Walking/stair climbing	Light	240′	4*3′ every 60′
Charlett et al. (2021)*, UK	Crossover	12	58	25.0 (6.0)	Psychomotor skills, attention, executive function, memory	Bodyweight resistance	not specified	300′	8*3′ every 30′
Chrismas et al. (2019)*, Qatar	Crossover	11	100	27.0** (−)	Memory, attention, executive function	Walking	Moderate	300′	9*3′ every 30′
Giurgiu et al. (2024), Germany	Encouragement approach	211	55	35.6 (10.6)	Executive function	Walking	Light-to-moderate	480′	3*3′ after 30′
Heiland et al. (2021), Sweden	Crossover	13	39	50.5 (4.6)	Executive function	Walking/bodyweight resistance	Light	180′	6*3′ every 30′
Horiuchi et al. (2023), Japan	Crossover	20	45	21.0 (1.0)	Executive function	Half-squats	not specified	180′	9*1′ every 20′
Kuo et al. (2024), Taiwan	Crossover	24	46	31.0 (8.0)	Executive function	Walking	Light	320′	15*2′ every 20′
Niedermeier et al. (2020), Austria	Parallel-group	51	33	23.3 (2.0)	Executive function, attention	Running	Intense	98′	1*10′ after 45′
Sperlich et al. (2018)*, Germany	Crossover	12	58	22.0 (2.0)	Executive function	HIIT	Intense	236′	1*6′ after 60′
Thirunavukarasu et al. (2025), India	Crossover	26	58	24.1 (2.4)	Attention	Stair climbing	Moderate	180′	3*2′ every 30′
Vincent et al. (2018)*, Australia	Crossover	6	0	27.0 (3.7)	Attention, executive function	Walking	Light	five consecutive days and nights	17*3′ every 30′
Wu et al. (2023), Netherlands	Crossover	21	71	24.0 (3.0)	Attention, executive function	Walking	Light	240′	8*5′ every 25′
Yu et al. (2022), China	Parallel-group	60	0	C: 21.1 (1.9) I: 21.2 (2.2)	Memory	Cycling	Light	105′	1*15′ after 45′
Yu et al. (2025), China	Crossover	71	51	20.9 (1.9)	Executive function	Cycling	Moderate-to-intense	115′	1*15′ after 50′
Outcome: EEG and cognitive performance									
Chueh et al. (2025), Taiwan	Crossover	18	0	25.0 (4.0)	Executive function	Walking	Light	210′	7*3′ every 30′
Outcome: EMG									
Ding et al. (2020), China	Parallel-group	72	50	C: 23.7 (1.01) I: 22.8 (1.1)	-	Postural change and walking	not specified	120′	1*5′ or 10′ after 40′
McLean et al. (2001), Canada	Parallel-group	15	100	34** (−)	-	Walking	Light	Working day	0.5′ every 20′ or 40′

* Studies also identified in the review by Li et al. (2022); **median.

C = control, I = intervention, EEG, electroencephalography; EMG, electromyography.

The studies were published between 2001 and 2025. The sample size ranged from 6 to 211 participants with a mean age between 20 and 50 years. Three studies examined only men (Vincent et al., 2018; Chueh et al., 2022; Yu et al., 2022), while two studies examined only women (McLean et al., 2001; Chrismas et al., 2019). We identified only one study that considered EEG as an outcome (Chueh et al., 2025), and two that collected EMG data (McLean et al., 2001; Ding et al., 2020). Sixteen studies considered CP as an outcome (Bergouignan et al., 2016; Sperlich et al., 2018; Vincent et al., 2018; Chrismas et al., 2019; Niedermeier et al., 2020; Charlett et al., 2021; Heiland et al., 2021; Yu et al., 2022; Chandran et al., 2023; Horiuchi et al., 2023; Wu et al., 2023; Giurgiu et al., 2024; Kuo et al., 2024; Thirunavukarasu et al., 2024; Chueh et al., 2025; Yu et al., 2025).

Twelve studies examined the effects of walking or running as a form of PA following prolonged periods of sitting (McLean et al., 2001; Bergouignan et al., 2016; Vincent et al., 2018; Chrismas et al., 2019; Ding et al., 2020; Niedermeier et al., 2020; Heiland et al., 2021; Chandran et al., 2023; Wu et al., 2023; Giurgiu et al., 2024; Kuo et al., 2024; Chueh et al., 2025). Three studies examined the effects of cycling (Yu et al., 2022; Yu et al., 2025) and bodyweight exercises (Charlett et al., 2021; Heiland et al., 2021; Horiuchi et al., 2023). Two studies involved participants climbing stairs (Chandran et al., 2023; Thirunavukkarasu et al., 2025) and one completing high-intensity interval training (HIIT) (Sperlich et al., 2018). Five of the identified studies (Bergouignan et al., 2016; Sperlich et al., 2018; Vincent et al., 2018; Chrismas et al., 2019; Charlett et al., 2021) had also been reported in a meta-analysis of exercise breaks on cognitive functions (Li et al., 2022).

**FIGURE 2:**
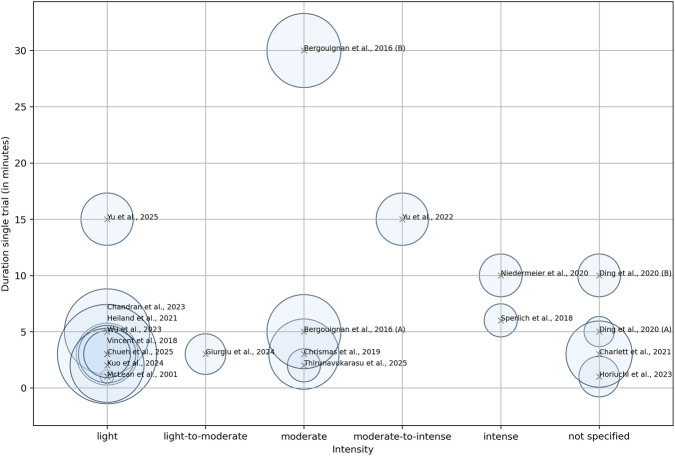
Bubble chart summarizing physical activity interventions across studies. x-axis = exercise intensity, y-axis = duration of a single trial (in minutes), bubble size = cumulative exposure duration. Studies labeled with (A) and (B) refer to distinct experimental conditions within the same publication.

The intensity of exposure ranged from light to intense activity; three studies did not specify the intensity (Ding et al., 2020; Charlett et al., 2021; Horiuchi et al., 2023). There was also considerable variation in the duration of the experiments and the length of active breaks from sitting. Experiments ranged from short laboratory visits lasting less than 2 hours to entire simulated working days lasting several days. Some studies involved taking sitting breaks of between five and 15 min once, or of between 0.5 and 8 minutes three to 17 times, throughout the day. A schematic bubble plot (Figure 2) provides an overview of the heterogeneity of the training protocols in the various studies, summarizing the most important methodological characteristics. It illustrates training intensity and the duration of a single trial, representing cumulative exposure duration based on bubble size. The length of time spent sitting before the respective physical activity break varied between 20 and 60 min.

### 3.3 Study quality

**FIGURE 3:**
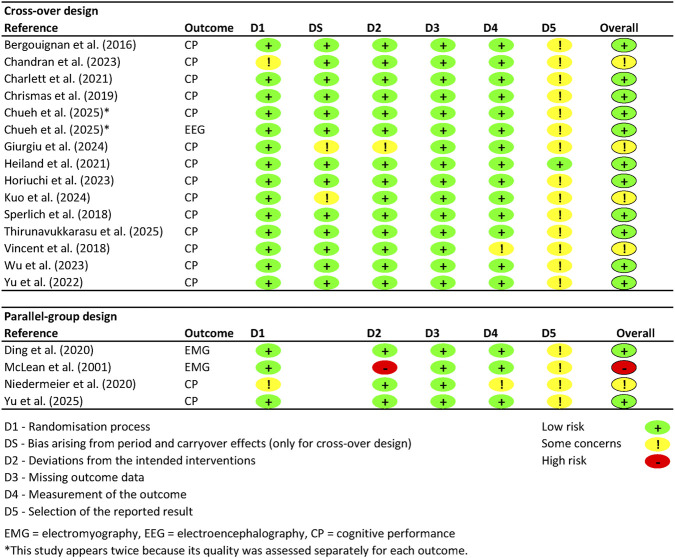
Quality assessment for each study using the Revised Cochrane Risk-of-Bias Tool for Randomised Trials (RoB 2 tool).

The results of the quality assessment using the RoB two tool (Sterne et al., 2019) are shown in Figure 3, differentiated by crossover and parallel-group designs and by outcome measure. Only one study had a published protocol, which allowed the quality dimension D5 *selection of the reported results* to be rated positively (Heiland et al., 2021). For the other studies, this information was unavailable. Nevertheless, the authors chose not to include this quality dimension in the overall assessment, noting that protocol publication remains unfortunately rare in this area of experimental research. One study was judged to be at *high risk* for the quality dimension *deviations from the intended interventions* due to vague instructions—participants were merely told to move away from their workstation for 30 s, with no mechanism to verify compliance (McLean et al., 2001). Studies were rated as having ‘some concerns’ when specific methodological weaknesses were identified—for example, if there has been no washout phase of at least 48 h between measurements (potentially leading to carry-over effects), if the exposure was not adequately controlled, or if the objectivity of outcome data collection was compromised. In total, 13 out of 19 studies were rated as having a *low risk of bias*, five raised *some concerns*, and one was rated as *high risk*.

### 3.4 Narrative synthesis

#### 3.4.1 Cognitive performance

Several of the included studies demonstrated beneficial effects of breaking up prolonged sitting with PA, particularly moderate-intensity activities such as walking or climbing stairs, on CP. Across these studies, participants were exposed to various break protocols, including light to vigorous walking, stair climbing, resistance exercises, and cycling with cognitive outcomes being assessed through tasks targeting attention, executive function, memory, and processing speed.

For example, breaking up sitting time with frequent 3-min moderate-intensity walking every 30 min significantly improved attention and executive function, as evidenced by faster reaction times in cognitive tasks such as Choice Reaction Time and Stroop (Chrismas et al., 2019). Brief half-squat exercises performed every 20 min during a 3-h sitting period have also been shown to enhance performance in the Stroop task and Trail Making Test, with moderate effect sizes (Horiuchi et al., 2023), while single 10-min walking breaks can positively influence visual attention (Niedermeier et al., 2020). Additionally, both moderate- and vigorous-intensity 15-min cycling breaks improved reaction time, error rate, and inverse efficiency in a dual-task Stroop paradigm, with the strongest effects observed under vigorous intensity (Yu et al., 2025).

Other studies have reported selective or modest cognitive benefits, often influenced by task type or timing. Taking moderate-intensity breaks by climbing stairs for 3 minutes has been shown to prevent deterioration in accuracy and reaction time during prolonged sitting (Chandran et al., 2023). No significant differences in CP were observed between light and moderate intensity. Conversely, interrupting sitting with light 5-min walking breaks has been found to improve task-switching performance in the Trail Making Test. However, it did not affect attention, inhibition or WM in other cognitive tasks (Wu et al., 2023). Positive effects on WM have also been observed following 3-min moderate walking breaks, whereas 3-min resistance training breaks did not yield similar benefits (Heiland et al., 2021).

Yet, several studies were unable to identify any significant cognitive improvements resulting from activity breaks. For instance, 3-min resistance training breaks did not yield measurable cognitive benefits (Charlett et al., 2021). Short HIIT sessions and frequent 3-min walking breaks showed no impact on Stroop task performance (Sperlich et al., 2018; Chueh et al., 2025), and a single 15-min cycling break failed to enhance episodic memory accuracy (Yu et al., 2022). No notable differences in task-switching ability, Trail Making Test results, or Flanker Test performance were observed across conditions (Bergouignan et al., 2016; Kuo et al., 2024). Ambulatory assessments also revealed no significant effects of walking breaks on WM (Giurgiu et al., 2024). Furthermore, frequent 3-min light walking breaks—even under sleep restriction—did not influence vigilance or WM (Vincent et al., 2018), and stair climbing was found to have no effect on attention compared to uninterrupted sitting (Thirunavukkarasu et al., 2025).

#### 3.4.2 Psychophysiological activity

Only one of our included studies investigated the effects of exercise, in this case light-intensity walking on a treadmill, on neurocognitive processes. A larger P3 amplitude, but no change in P3 latency, was observed in the active condition compared to the seated control group during a cognitive task (Chueh et al., 2025).

#### 3.4.3 Muscular parameters

Two studies have examined the physiological effects of interrupting prolonged sitting, each emphasizing muscular parameters measured by EMG but with different methodological approaches.

Changes in muscle fatigue under different break conditions in two back muscles have been examined (Ding et al., 2020). Muscle fatigue was detected after 40–50 min of sitting and was alleviated by various breaks, compared to the seated reference group who did not take breaks. However, the study contradicts itself in its statements about which type of break (changing posture and take a walk, or standing and stretching) was the most effective in alleviating muscle fatigue. Regarding the effectiveness of active break durations, there was reportedly no difference between 5- and 10-min breaks. In contrast, a field study provided a neurophysiological perspective by identifying an increase of a cyclic trend in the myoelectric signal of various muscles after 30 s activity breaks, compared to the control group (McLean et al., 2001). These cyclings are thought to result from changes in the recruitment of different motor units to maintain static postures, as well as the body’s own mechanisms for preventing muscle fatigue.

## 4 Discussion

### 4.1 Explanation of results

The aim of the study was to investigate the effect of active breaks during prolonged sitting on CP and muscle strain in healthy adults of working age. In particular, the study sought to answer the question of the extent to which the type, duration, and frequency of the intervention influenced the results. The results of the individual studies appear to differ greatly and depend on various factors. Firstly, the outcome measured and how it is measured play a role.

Regarding the EMG measurements, two different approaches were employed to examine the impact of activity breaks. These were fatigue while sitting and after the movement break, and changes in cyclical muscle activity fluctuations depending on the condition. Both studies concluded that movement breaks have a positive effect on muscle health. Ding et al. (2020) demonstrated that prolonged sitting for approximately 40 min leads to fatigue in the back muscles, which can be alleviated by exercise breaks. This finding is important for designing work and determining the optimal timing of short exercise breaks. McLean et al. (2001) interpret the increase in cycling trends after activity breaks as a positive effect, meaning that muscle fatigue can be prevented more effectively by recruiting different motor units to maintain static postures. One limitation is that the study design apparently does not control whether participants actually performed the active movement. Although they were prompted by a computer program to leave their workstations for 30 s after 20- or 40-min intervals, there was no control or verification to ensure that they actually complied with this request. This could compromise the validity of the results regarding the effectiveness of exercise breaks. This led us to a high-risk assessment of the quality. In summary, it can be said that the body has mechanisms to counteract muscle fatigue, but these cannot be maintained indefinitely. However, active breaks seem to help keep these mechanisms active and thus prevent an increase in cardiovascular risk markers. However, the small number of studies does not allow for reliable evidence.

Although only one EEG study could be included, its findings reveal a positive effect of regular light walking breaks on the neurocognitive functions of young men (Chueh et al., 2025). Interestingly, no improvements in inhibitory control were observed. However, that absence underscores the limitations of studies that rely solely on behavioural data to assess cognitive change, as subtle but meaningful changes in brain activity may be overlooked. Neurophysiological assessments, such as EEG, offer a more sensitive lens for detecting cognitive shifts that remain hidden in traditional performance-based tests, and should therefore be considered a necessary complement in future research designs.

In other words, changes in brain activity may occur despite stable response times or accuracy, indicating early cognitive adaptation. Studies that examine the influence of single bouts of exercise on neurocognitive functions in general, rather than the effect of sitting breaks, have come to similar conclusions. For instance, P300 amplitude in college soccer players performing the Stroop task was higher following a 30-min treadmill session and 30 min of futsal than in the seated control group (Won et al., 2017). However, there was also an impact on inhibitory control, evidenced by a reduction in reaction time in the Stroop task. Similarly, during a Flanker task, reaction time was reduced and P300 increased after a treadmill session compared to the seated control group (O’Leary et al., 2011). Other studies found no improvement in cognitive processes following individual exercise breaks, but a prevention of attentional decrements, which were observed during periods of sitting without exercise breaks (Pontifex et al., 2015; Kao et al., 2022a).

The varying effects of the interventions on CP appear to depend in part on an interaction between the type of cognitive test - thus the cognitive domain - and the intensity, duration, and type of intervention. The results suggest that moderate activity breaks in particular have a positive effect on WM. Breaks that are too short, too infrequent or with low intensity seem to have a less positive effect on CP. The timing of the measurement after a break in activity can also play a role. In the studies included, CP was often measured at only one or two points in time, at the beginning and/or only at the end of the experiment (Bergouignan et al., 2016; Niedermeier et al., 2020; Charlett et al., 2021; Heiland et al., 2021; Yu et al., 2022; Horiuchi et al., 2023; Wu et al., 2023; Kuo et al., 2024; Chueh et al., 2025; Yu et al., 2025). This is advantageous in order to minimize learning effects in the cognitive tasks, but makes it more difficult to gain accurate insights into the short-term effects. If the measurement is taken too long after the intervention, the positive effect may have already disappeared, regardless of the intensity of the activity, and a renewed negative effect of sedentary and mental stress may occur (Larson et al., 2024). Nevertheless, reducing sedentary behaviour by increasing PA appears to have long-term positive effects on executive functions (Liu et al., 2024). Furthermore, interindividual differences in physical fitness level or cognitive baseline can influence the size and even the presence of cognitive benefits (Schwarck et al., 2019; Cui et al., 2020). In addition, interindividual differences in the inability to recover may influence CP following breaks that involve PA. Recovery processes aim to reduce both physiological and psychological stress responses caused by work demands to baseline levels, typically occurring during or outside working hours (Geurts and Sonnentag, 2006). Inadequate recovery, often due to a low capacity for psychological detachment from work despite adequate break time, impairs regeneration from stress and is associated with long-term health risks (Geurts and Sonnentag, 2006; Wendsche and Lohmann-Haislah, 2016; Richter et al., 2017; Wendsche et al., 2018). As a result, identical interventions, such as short exercise breaks, may have varying effects depending on the individual’s inability to recover. Although this was not the focus of this review, it seems that the inability to recover has not been widely investigated as a moderating factor to date, and should be addressed in future research.

Another factor to consider for the heterogeneous results on cognitive outcomes may be the tasks assigned during the sitting sessions. In most studies, participants were free to choose whether they wanted to read, watch TV, talk, work on the computer, or send messages. While two of the studies took place in real work environments and during regular working hours (McLean et al., 2001; Giurgiu et al., 2024), two other studies employed standardised and realistic work tasks to keep participants occupied during the sitting phases (Ding et al., 2020; Thirunavukkarasu et al., 2025). Thus, the mental load varies during the different tasks, which in turn can affect the CP results after the interventions (Wanders et al., 2021). The effect of the intervention may not be solely attributed to the exercise, as it could be confounded by varying levels of mental (in)activity during the sitting phase. Future studies should make greater use of standardized and cognitively comparable tasks in order to more clearly determine the effect of exercise breaks on CP.

Further potential sources of variability across the included studies are the differing durations of the sitting periods prior to activity breaks. While our review did not specify a minimum sitting time, the length of the preceding sedentary period may affect the intensity of the psychophysiological and muscular responses. Shorter sitting periods may not induce sufficient cognitive fatigue, mental load or muscular strain to produce measurable changes following a movement break. In contrast, longer periods of uninterrupted sitting could amplify these effects, thereby increasing the likelihood of detecting benefits. Therefore, inconsistencies in the reported outcomes may be partly explained by heterogeneity in pre-activity sitting durations and should be considered when interpreting the findings.

The results included in this scoping review are subject to several limitations that should be considered when interpreting the findings. First, most included studies had small samples, which limits statistical power and increases the risk of chance findings. Second, the interventions were typically short-term, providing limited insight into long-term cognitive effects of activity breaks. Third, there was considerable heterogeneity in the cognitive tasks used (e.g., Stroop, Flanker, Trail Making, n-Back) and thus in the cognitive domains, which makes it difficult to compare the results. Another issue lies in untested baseline differences between the various measurement days in cross-over designs. For example, in Supplementary Material *four* by Chandran et al. (2023), differences prevailing at the first measurement time of each measurement day can be inferred, but no testing of these differences for significance is reported. In addition, some studies measured cognitive functions only once after the intervention, which means that the temporal course of attention processes in response to prolonged sitting and intermittent activity breaks cannot be determined (Bergouignan et al., 2016; Chrismas et al., 2019; Charlett et al., 2021; Chueh et al., 2022; Yu et al., 2022; Horiuchi et al., 2023).

The results of the review and the limitations of the included studies underscore the need for more robust, ecologically valid, and longitudinal studies with larger samples to better understand the cognitive and health effects of interrupting sedentary activities during work.

Despite their methodological differences and limitations, the combined findings support the implementation of regular, even brief, PA breaks to enhance both CP and muscular health. Even though the studies come to different conclusions, the few positive results regarding improved health and CP alone justify to reduce sedentary time in accordance with WHO guidelines (World Health Organization, 2020) and increasing PA at the same time. This is particularly important in the workplace, where people spend most of their time sitting.

### 4.2 Strengths and limitations

The review is based on a systematic exploration of the currently available research, ensuring that the included studies reflect the most recent findings and trends relevant to active breaks during prolonged sitting. Unlike previous reviews, it integrates a multi-level outcome perspective by examining neurophysiological, cognitive, and muscular domains. This comprehensive approach enables a broader understanding of the effects of active breaks. Furthermore, the review places a particular emphasis on the methodological diversity of experimental designs and examines whether differences in the type, duration, frequency, and measurement methods of interventions may contribute to the heterogeneity of reported results. This focus might improve interpretability and provides a basis for future standardization of study protocols. A formal evaluation of the risk of bias was conducted across all included studies. This methodological rigor enhances the credibility and interpretability of the findings, providing readers with greater transparency regarding study quality and internal validity. By highlighting existing gaps and inconsistencies, the review paves the way for more targeted and robust studies in this field.

Nevertheless, this review has some limitations. First, only studies involving healthy adults of working age were included, deliberately excluding older people and those with pre-existing conditions or overweight-related health risks. Therefore, the generalizability of the results to vulnerable populations, such as elderly individuals or those with chronic conditions, remains limited. Second, due to the methodological heterogeneity among the included studies, particularly regarding intervention type, duration, frequency, and assessment methods, a quantitative meta-analysis was not feasible. Consequently, the findings were summarised using a narrative approach, which limits direct comparisons and restricts the derivation of aggregate effect sizes. Fourth, due to the lack of standardised study protocols, we were unable to reliably assess the risk of bias for selection of the reported results.

## 5 Conclusion

This scoping review examined the impact of active breaks on CP and muscle strain in healthy working-age adults during prolonged sitting. It highlights considerable differences between individual studies, mainly due to variations in intervention type, duration, frequency and timing of measurements. Synthesising neurophysiological, muscular and cognitive results provides promising evidence that regular exercise breaks, even if they are short, can reduce muscle fatigue and support cognitive function, particularly in areas such as WM. The promising role of neurophysiological methods, such as EEG in detecting subtle cognitive shifts that are not always reflected in the results of behavioural tests underscores the potential of this research field. Nevertheless, the body of research in this area remains insufficient. The limited number of studies, small sample sizes and methodological heterogeneity - particularly with regard to the design of cognitive tasks and ecological validity - complicate the interpretation of findings. Factors such as non-standardised mental tasks during sitting phases and the timing of cognitive assessments also influence variability in the results. Due to these limitations, the review provides some evidence that active breaks after sedentary time may contribute to maintaining cognitive performance and muscle health. These findings highlight the need for more robust and ecologically valid research.

## Data Availability

The contributions excluded from the study are included in the Supplementary Material, further inquiries can be directed to the corresponding author.

## References

[B1] Akkarakittichoke N. Waongenngarm P. Janwantanakul P. (2021). The effects of active break and postural shift interventions on recovery from and recurrence of neck and low back pain in office workers: a 3-arm cluster-randomized controlled trial. Musculoskelet. Sci. Pract. 56, 102451. 10.1016/j.msksp.2021.102451 34450361

[B2] Barlow C. E. Shuval K. Balasubramanian B. A. Kendzor D. E. Radford N. B. DeFina L. F. (2016). Association between sitting time and cardiometabolic risk factors after adjustment for cardiorespiratory fitness, cooper center longitudinal study, 2010-2013. Prev. Chronic Dis. 13, E181. 10.5888/pcd13.160263 28033088 PMC5201150

[B3] Bergouignan A. Legget K. T. Jong N. de Kealey E. Nikolovski J. Groppel J. L. (2016). Effect of frequent interruptions of prolonged sitting on self-perceived levels of energy, mood, food cravings and cognitive function. Int. J. Behav. Nutr. Phys. Act. 13, 113. 10.1186/s12966-016-0437-z 27809874 PMC5094084

[B4] Brakenridge C. J. Koster A. Galan B. E. de Carver A. Dumuid D. Dzakpasu F. Q. S. (2024). Associations of 24 h time-use compositions of sitting, standing, physical activity and sleeping with optimal cardiometabolic risk and glycaemic control: the maastricht study. Diabetologia 67, 1356–1367. 10.1007/s00125-024-06145-0 38656371 PMC11153304

[B5] Chambers A. J. Robertson M. M. Baker N. A. (2019). The effect of sit-stand desks on office worker behavioral and health outcomes: a scoping review. Appl. Ergon. 78, 37–53. 10.1016/j.apergo.2019.01.015 31046958

[B6] Chandran O. Shruthi P. Sukumar S. Kadavigere R. Chakravarthy K. Rao C. R. (2023). Effects of physical activity breaks during prolonged sitting on vascular and executive function-A randomised cross-over trial. J. Taibah Univ. Med. Sci. 18, 1065–1075. 10.1016/j.jtumed.2023.03.004 36994221 PMC10040888

[B7] Chandrasekaran B. Pesola A. J. Rao C. R. Arumugam A. (2021). Does breaking up prolonged sitting improve cognitive functions in sedentary adults? A mapping review and hypothesis formulation on the potential physiological mechanisms. BMC Musculoskelet. Disord. 22, 274. 10.1186/s12891-021-04136-5 33711976 PMC7955618

[B8] Chang Y. K. Labban J. D. Gapin J. I. Etnier J. L. (2012). The effects of acute exercise on cognitive performance: a meta-analysis. Brain Res. 1453, 87–101. 10.1016/j.brainres.2012.02.068 22480735

[B9] Charlett O. P. Morari V. Bailey D. P. (2021). Impaired postprandial glucose and no improvement in other cardiometabolic responses or cognitive function by breaking up sitting with bodyweight resistance exercises: a randomised crossover trial. J. Sports Sci. 39, 792–800. 10.1080/02640414.2020.1847478 33213284

[B10] Chastin S. F. M. Craemer M. de Cocker K. de Powell L. van Cauwenberg J. Dall P. (2019). How does light-intensity physical activity associate with adult cardiometabolic health and mortality? Systematic review with meta-analysis of experimental and observational studies. Br. J. Sports Med. 53, 370–376. 10.1136/bjsports-2017-097563 29695511 PMC6579499

[B11] Chaudhary N. Jones M. Rice S. P. M. Zeigen L. Thosar S. S. (2024). Transitioning to working from home due to the COVID-19 pandemic significantly increased sedentary behavior and decreased physical activity: a meta-analysis. Int. J. Environ. Res. Public Health 21, 851. 10.3390/ijerph21070851 39063428 PMC11276674

[B12] Chrismas B. C. R. Taylor L. Cherif A. Sayegh S. Bailey D. P. (2019). Breaking up prolonged sitting with moderate-intensity walking improves attention and executive function in Qatari females. PLoS One 14, e0219565. 10.1371/journal.pone.0219565 31299061 PMC6625720

[B13] Chueh T.-Y. Chen Y.-C. Hung T.-M. (2022). Acute effect of breaking up prolonged sitting on cognition: a systematic review. BMJ Open 12, e050458. 10.1136/bmjopen-2021-050458 35292487 PMC8928248

[B14] Chueh T. Y. Chen Y. C. Hung T. M. (2025). Breaking up sitting enhances neurocognitive function which is associated with improved postprandial glucose regulation in healthy adults: a randomized crossover study. Physiol. Behav. 290, 114744. 10.1016/j.physbeh.2024.114744 39579950

[B15] Cui J. Zou L. Herold F. Yu Q. Jiao C. Zhang Y. (2020). Does cardiorespiratory fitness influence the effect of acute aerobic exercise on executive function? Front. Hum. Neurosci. 14, 569010. 10.3389/fnhum.2020.569010 33132882 PMC7573667

[B16] Dang T. H. an Starke K. R. Liebers F. Burr H. Seidler A. Hegewald J. (2024). Impact of sitting at work on musculoskeletal complaints of German workers - results from the study on mental health at work (S-MGA). J. Occup. Med. Toxicol. 19, 9. 10.1186/s12995-024-00408-7 38539214 PMC10967152

[B17] Ding Y. Cao Y. Duffy V. G. Zhang X. (2020). It is time to have rest: how do break types affect muscular activity and perceived discomfort during prolonged sitting work. Saf. Health Work 11, 207–214. 10.1016/j.shaw.2020.03.008 32596017 PMC7303538

[B18] Dzakpasu F. Q. S. Owen N. Carver A. Brakenridge C. J. Eakin E. G. Healy G. N. (2023). Changes in desk-based workers’ sitting, standing, and stepping time: short- and longer-term effects on musculoskeletal pain. Med. Sci. Sports Exerc 55, 2241–2252. 10.1249/MSS.0000000000003248 37729188

[B19] Ekelund U. Tarp J. Steene-Johannessen J. Hansen B. H. Jefferis B. Fagerland M. W. (2019). Dose-response associations between accelerometry measured physical activity and sedentary time and all cause mortality: systematic review and harmonised meta-analysis. BMJ 366, l4570. 10.1136/bmj.l4570 31434697 PMC6699591

[B20] Ekelund U. Tarp J. Fagerland M. W. Johannessen J. S. Hansen B. H. Jefferis B. J. (2020). Joint associations of accelerometer-measured physical activity and sedentary time with all-cause mortality: a harmonised meta-analysis in more than 44 000 middle-aged and older individuals. Br. J. Sports Med. 54, 1499–1506. 10.1136/bjsports-2020-103270 33239356 PMC7719907

[B21] Geurts S. A. E. Sonnentag S. (2006). Recovery as an explanatory mechanism in the relation between acute stress reactions and chronic health impairment. Scand. J. Work Environ. Health 32, 482–492. 10.5271/sjweh.1053 17173204

[B22] Giurgiu M. Timm I. Ebner-Priemer U. W. Schmiedek F. Neubauer A. B. (2024). Causal effects of sedentary breaks on affective and cognitive parameters in daily life: a within-person encouragement design. Npj Ment. Health Res. 3, 64. 10.1038/s44184-024-00113-7 39706901 PMC11662072

[B23] Heiland E. G. Tarassova O. Fernström M. English C. Ekblom Ö. Ekblom M. M. (2021). Frequent, short physical activity breaks reduce prefrontal cortex activation but preserve working memory in middle-aged adults: ABBaH study. Front. Hum. Neurosci. 15, 719509. 10.3389/fnhum.2021.719509 34602995 PMC8481573

[B24] Horiuchi M. Pomeroy A. Horiuchi Y. Stone K. Stoner L. (2023). Effects of intermittent exercise during prolonged sitting on executive function, cerebrovascular, and psychological response: a randomized crossover trial. J. Appl. Physiol. 135, 1421–1430. 10.1152/japplphysiol.00437.2023 37942532 PMC12088687

[B25] Kao S. C. Baumgartner N. Nagy C. Fu H. L. Yang C. T. Wang C. H. (2022a). Acute effects of aerobic exercise on conflict suppression, response inhibition, and processing efficiency underlying inhibitory control processes: an ERP and SFT study. Psychophysiology 59, e14032. 10.1111/psyp.14032 35199340

[B26] Kao S. C. Chen F. T. Moreau D. Drollette E. S. Amireault S. Chu C. H. (2022b). Acute effects of exercise engagement on neurocognitive function: a systematic review and meta-analysis on P3 amplitude and latency. Int. Rev. Sport Exerc. Psychol. 18, 1–43. 10.1080/1750984X.2022.2155488

[B27] Kar G. Hedge A. (2020). Effects of a sit-stand-walk intervention on musculoskeletal discomfort, productivity, and perceived physical and mental fatigue, for computer-based work. Int. J. Industrial Ergonomics 78, 102983. 10.1016/j.ergon.2020.102983 32818838

[B28] Kardys C. Küper K. Getzmann S. Falkenstein M. Voelcker-Rehage C. (2022). A comparison of the effects of short-term physical and combined multi-modal training on cognitive functions. Int. J. Environ. Res. Public Health 19 (12), 7506. 10.3390/ijerph19127506 35742756 PMC9223650

[B29] Kuo F. C. Lin Y. T. Chueh T. Y. Chang Y. K. Hung T. M. Chen Y. C. (2024). Breaking prolonged sitting increases 24-h physical activity and self-perceived energy levels but does not acutely affect cognition in healthy adults. Eur. J. Appl. Physiol. 124, 445–455. 10.1007/s00421-023-05278-1 37543544

[B30] Larson M. J. Muir A. M. Reid R. O. Carbine K. A. Marsh H. LaCouture H. (2024). Does intensity matter? A randomized crossover study of the role of acute exercise intensity on cognitive performance and motor speed and accuracy. Prog. Brain Res. 283, 99–121. 10.1016/bs.pbr.2024.01.001 38538194

[B31] Li J. Herold F. Ludyga S. Yu Q. Zhang X. Zou L. (2022). The acute effects of physical exercise breaks on cognitive function during prolonged sitting: the first quantitative evidence. Complement. Ther. Clin. Pract. 48, 101594. 10.1016/j.ctcp.2022.101594 35483298

[B32] Liu J. Wei M. Li X. Ablitip A. Zhang S. Ding H. (2024). Substitution of physical activity for sedentary behaviour contributes to executive function improvement among young adults: a longitudinal study. BMC Public Health 24, 3326. 10.1186/s12889-024-20741-0 39614217 PMC11605965

[B33] McLean L. Tingley M. Scott R. N. Rickards J. (2001). Computer terminal work and the benefit of microbreaks. Appl. Ergon. 32, 225–237. 10.1016/s0003-6870(00)00071-5 11394463

[B34] Miller K. R. McClave S. A. Jampolis M. B. Hurt R. T. Krueger K. Landes S. (2016). The health benefits of exercise and physical activity. Curr. Nutr. Rep. 5, 204–212. 10.1007/s13668-016-0175-5

[B35] Morgan R. L. Whaley P. Thayer K. A. Schünemann H. J. (2018). Identifying the PECO: a framework for formulating good questions to explore the association of environmental and other exposures with health outcomes. Environ. International 121, 1027–1031. 10.1016/j.envint.2018.07.015 30166065 PMC6908441

[B36] Nakphet N. Chaikumarn M. Janwantanakul P. (2014). Effect of different types of rest-break interventions on neck and shoulder muscle activity, perceived discomfort and productivity in symptomatic VDU operators: a randomized controlled trial. Int. J. Occup. Saf. Ergon. 20, 339–353. 10.1080/10803548.2014.11077048 24934429

[B37] Niedermeier M. Weiss E. M. Steidl-Müller L. Burtscher M. Kopp M. (2020). Acute effects of a short bout of physical activity on cognitive function in sport students. Int. J. Environ. Res. Public Health 17. 10.3390/ijerph17103678 32456170 PMC7277588

[B38] Osama M. Jan M. B. A. Darain H. (2015). A randomized controlled trial comparing the effects of rest breaks and exercise breaks in reducing musculoskeletal discomfort. Ann. Allied Health Sci. 1, 30–34. Available online at: https://aahs.kmu.edu.pk/index.php/aahs/article/view/46 .

[B39] Ouzzani M. Hammady H. Fedorowicz Z. Elmagarmid A. (2016). Rayyan-a web and Mobile app for systematic reviews. Syst. Rev. 5, 210. 10.1186/s13643-016-0384-4 27919275 PMC5139140

[B40] O’Leary K. C. Pontifex M. B. Scudder M. R. Brown M. L. Hillman C. H. (2011). The effects of single bouts of aerobic exercise, exergaming, and videogame play on cognitive control. Clin. Neurophysiol. 122, 1518–1525. 10.1016/j.clinph.2011.01.049 21353635

[B41] Patterson R. McNamara E. Tainio M. Sá T. H. de Smith A. D. Sharp S. J. (2018). Sedentary behaviour and risk of all-cause, cardiovascular and cancer mortality, and incident type 2 diabetes: a systematic review and dose response meta-analysis. Eur. J. Epidemiol. 33, 811–829. 10.1007/s10654-018-0380-1 29589226 PMC6133005

[B42] Polspoel M. Mullie P. Reilly T. van Tiggelen D. Calders P. (2025). Comparison of physical activity and sedentary behavior between telework and office work in a working population during the COVID-19 pandemic: a systematic review and meta-analysis of observational studies. BMC Public Health 25, 1805. 10.1186/s12889-025-22948-1 40380300 PMC12083111

[B43] Pontifex M. B. Parks A. C. Henning D. A. Kamijo K. (2015). Single bouts of exercise selectively sustain attentional processes. Psychophysiology 52, 618–625. 10.1111/psyp.12395 25523887 PMC4398582

[B44] Prince S. A. Elliott C. G. Scott K. Visintini S. Reed J. L. (2019). Device-measured physical activity, sedentary behaviour and cardiometabolic health and fitness across occupational groups: a systematic review and meta-analysis. Int. J. Behav. Nutr. Phys. Act. 16, 30. 10.1186/s12966-019-0790-9 30940176 PMC6444868

[B45] Prince S. A. Cardilli L. Reed J. L. Saunders T. J. Kite C. Douillette K. (2020). A comparison of self-reported and device measured sedentary behaviour in adults: a systematic review and meta-analysis. Int. J. Behav. Nutr. Phys. Act. 17, 31. 10.1186/s12966-020-00938-3 32131845 PMC7055033

[B46] Proper K. I. Singh A. S. van Mechelen W. Chinapaw M. J. M. (2011). Sedentary behaviors and health outcomes among adults: a systematic review of prospective studies. Am. J. Prev. Med. 40, 174–182. 10.1016/j.amepre.2010.10.015 21238866

[B47] Reiner M. Niermann C. Jekauc D. Woll A. (2013). Long-term health benefits of physical activity—a systematic review of longitudinal studies. BMC Public Health 13, 813. 10.1186/1471-2458-13-813 24010994 PMC3847225

[B48] Richter P. Funke C. Mittmann S. Rudolf M. Zwingmann I. (2017). Gesundheitsrelevante Beeinflussung der Handlungsregulation unter psychischer Belastung – entwicklung von Parallelskalen zum FABA-Fragebogen [Health-related modification of action regulation under stress - development of parallel scales of the faulty attitudes and behavior analysis questionnaire]. Psychol. Des. Alltagshandelns 10, 5–18. Available online at: https://www.allgemeine-psychologie.info/cms/images/stories/allgpsy_journal/Vol%2010%20No%201/01_abstract_richter.pdf .

[B49] Sauter M. Backé E. Pfab C. Prigge M. Brendler C. Liebers F. (2025). Comparison of sedentary time, number of steps and sit-to-stand-transitions of desk-based workers in different office environments including working from home: analysis of quantitative accelerometer data from the cross-sectional part of the SITFLEX Study. Scand. J. Work Environ. Health 51, 333–343. 10.5271/sjweh.4228 40273437 PMC12282600

[B50] Schmidt N. Romero Starke K. Sauter M. Burr H. Seidler A. Hegewald J. (2025). Sitting time at work and cardiovascular disease risk-a longitudinal analysis of the Study on Mental Health at work (S-MGA). Int. Arch. Occup. Environ. Health 98, 119–133. 10.1007/s00420-024-02118-3 39841190 PMC11807066

[B51] Scholz A. Ghadiri A. Singh U. Wendsche J. Peters T. Schneider S. (2018). Functional work breaks in a high-demanding work environment: an experimental field study. Ergonomics 61, 255–264. 10.1080/00140139.2017.1349938 28679350

[B52] Schöne C. Sauter M. Backé E.-M. Prigge M. Brendler C. Hegewald J. (2025). The impact of working from home on sedentary behaviour and physical activity compared to onsite work in the working population: a systematic review and meta-analysis. BMC Public Health 25, 3963. 10.1186/s12889-025-24960-x 41249967 PMC12621373

[B53] Schwarck S. Schmicker M. Dordevic M. Rehfeld K. Müller N. Müller P. (2019). Inter-Individual differences in cognitive response to a single bout of physical Exercise-A randomized controlled cross-over Study. J. Clin. Med. 8, 1101. 10.3390/jcm8081101 31349593 PMC6723732

[B54] Sortino M. Trovato B. Zanghì M. Roggio F. Musumeci G. (2024). Active breaks reduce back overload during prolonged sitting: ergonomic analysis with infrared thermography. J. Clin. Med. 13, 3178. 10.3390/jcm13113178 38892891 PMC11172579

[B55] Sousa Fernandes M. S. de Ordônio T. F. Santos G. C. J. Santos L. E. R. Calazans C. T. Gomes D. A. (2020). Effects of physical exercise on neuroplasticity and brain function: a systematic review in human and animal studies. Neural Plast. 2020, 8856621. 10.1155/2020/8856621 33414823 PMC7752270

[B56] Sperlich B. Clerck I. de Zinner C. Holmberg H. C. Wallmann-Sperlich B. (2018). Prolonged sitting interrupted by 6-Min of high-intensity exercise: circulatory, metabolic, hormonal, thermal, cognitive, and perceptual responses. Front. Physiol. 9, 1279. 10.3389/fphys.2018.01279 30386249 PMC6198043

[B57] Sterne J. A. C. Savović J. Page M. J. Elbers R. G. Blencowe N. S. Boutron I. (2019). RoB 2: a revised tool for assessing risk of bias in randomised trials. BMJ 366, l4898. 10.1136/bmj.l4898 31462531

[B58] Sudo M. Costello J. T. McMorris T. Ando S. (2022). The effects of acute high-intensity aerobic exercise on cognitive performance: a structured narrative review. Front. Behav. Neurosci. 16, 957677. 10.3389/fnbeh.2022.957677 36212191 PMC9538359

[B59] Thirunavukarasu E. T. Reddy M. Chandrasekaran B. Maiya A. G. Rao C. R. (2024). Stair climbing interventions reduce postprandial hyperglycemia but not cognitive functions: findings of a randomized cross-over trial. Physiol. Behav., 114726. 10.1016/j.physbeh.2024.114726 39489460

[B60] Thirunavukkarasu E. Aerva M. R. Chandrasekaran B. Maiya G. A. Rao C. R. (2025). Short-term effects of brief stair climbing interruptions on postprandial hyperglycemia during prolonged sitting: a randomized cross-over trial. Sci. Rep. 15, 2329. 10.1038/s41598-024-77827-3 39824875 PMC11742412

[B61] Vincent G. E. Jay S. M. Sargent C. Kovac K. Vandelanotte C. Ridgers N. D. (2018). The impact of breaking up prolonged sitting on glucose metabolism and cognitive function when sleep is restricted. Neurobiol. Sleep. Circadian Rhythms 4, 17–23. 10.1016/j.nbscr.2017.09.001 31236503 PMC6584591

[B62] Wanders L. Bakker E. A. van Hout H. P. J. Eijsvogels T. M. H. Hopman M. T. E. Visser L. N. C. (2021). Association between sedentary time and cognitive function: a focus on different domains of sedentary behavior. Prev. Med. 153, 106731. 10.1016/j.ypmed.2021.106731 34280406

[B63] Waongenngarm P. van der Beek A. J. Akkarakittichoke N. Janwantanakul P. (2020). Perceived musculoskeletal discomfort and its association with postural shifts during 4-h prolonged sitting in office workers. Appl. Ergon. 89, 103225. 10.1016/j.apergo.2020.103225 32755740

[B64] Wendsche J. Lohmann-Haislah A. (2016). A meta-analysis on antecedents and outcomes of detachment from work. Front. Psychol. 7, 2072. 10.3389/fpsyg.2016.02072 28133454 PMC5233687

[B65] Wendsche J. Lohmann-Haislah A. Schulz A. Schoellgen I. (2018). Mentales Abschalten von der Arbeit als Erholungsindikator: Wirkungen, Einflussfaktoren und Gestaltungsansätze Mental detachment from work as an indicator of recovery: outcomes, antecedents and associated interventions. ASU Arbeitsmed Sozialmed Umweltmed, 25–31. Available online at: https://www.asu-arbeitsmedizin.com/mentales-abschalten-als-erholungsindikation/mentales-abschalten-von-der-arbeit-als .

[B66] Wennberg P. Boraxbekk C.-J. Wheeler M. Howard B. Dempsey P. C. Lambert G. (2016). Acute effects of breaking up prolonged sitting on fatigue and cognition: a pilot study. BMJ Open 6, e009630. 10.1136/bmjopen-2015-009630 26920441 PMC4769400

[B67] Wilmot E. G. Edwardson C. L. Achana F. A. Davies M. J. Gorely T. Gray L. J. (2012). Sedentary time in adults and the association with diabetes, cardiovascular disease and death: systematic review and meta-analysis. Diabetologia 55, 2895–2905. 10.1007/s00125-012-2677-z 22890825

[B68] Wollseiffen P. Ghadiri A. Scholz A. Strüder H. K. Herpers R. Peters T. (2016). Short bouts of intensive exercise during the workday have a positive effect on Neuro-cognitive performance. Stress Health 32, 514–523. 10.1002/smi.2654 26449710

[B69] Won J. Wu S. Ji H. Smith J. C. Park J. (2017). Executive function and the P300 after treadmill exercise and futsal in college soccer players. Sports (Basel) 5. 10.3390/sports5040073 29910433 PMC5969040

[B70] World Health Organization (2020). WHO Guidelines on physical activity and sedentary behaviour. Geneva: World Health Organization. 10.1136/bjsports-2020-102955 PMC7719906 33239350

[B71] Wu Y. van Gerven P. W. M. Groot R. H. M. de Eijnde B. O. Winkens B. Savelberg H. (2023). Effects of breaking up sitting with light-intensity physical activity on cognition and mood in university students. Scand. J. Med. Sci. Sports 33, 257–266. 10.1111/sms.14277 36434768

[B72] Yu Q. Herold F. Ludyga S. Cheval B. Zhang Z. Mücke M. (2022). Neurobehavioral mechanisms underlying the effects of physical exercise break on episodic memory during prolonged sitting. Complement. Ther. Clin. Pract. 48, 101553. 10.1016/j.ctcp.2022.101553 35395497

[B73] Yu Q. Zhang Z. Ludyga S. Erickson K. I. Cheval B. Hou M. (2025). Effects of physical exercise breaks on executive function in a simulated classroom setting: uncovering a window into the brain. Adv. Sci. (Weinh) 12, e2406631. 10.1002/advs.202406631 39584316 PMC11744571

