# Supplementary material for: Evaluating the methods used to examine sitting breaks and their influence on mental load, physical strain, and cognitive performance - a scoping review
Source: Front Physiol. 2026 Feb 18;17:1755356. doi: 10.3389/fphys.2026.1755356 (PMC12956524; doi:10.3389/fphys.2026.1755356)
Supplement: Supplementary file 1 [file Table1.docx]

**S1 Table:** **Overview of the excluded studies.**

| **Authors (year)** | **Title** | **Journal** | **Reason for**  **exclusion** |
| --- | --- | --- | --- |
| Alghadier, M.; Alharbi, T.; Almasoud, N. & Alshalawi, A. A.  (2024) | Active Video Games Using Virtual Reality Influence Cognitive Performance in Sedentary Female University Students: A Randomized Clinical Trial. | Life | Wrong exposure |
| Altenburg, T. M.; Rotteveel, J.; Dunstan, D. W.; Salmon, J. & Chinapaw, M. J. (2013) | The effect of interrupting prolonged sitting time with short, hourly, moderate-intensity cycling bouts on cardiometabolic risk factors in healthy, young adults | Journal of applied physiology | Wrong outcome |
| Bailey, A. W.; Kang, H. K.  (2022) | Walking and Sitting Outdoors: Which Is Better for Cognitive Performance and Mental States? | International journal of environmental research and public health | Wrong exposure |
| Bailey, B. W.; Muir, A. M.; Bartholomew, C. L.; Christensen, W. F.; Carbine, K. A.; Marsh, H.; LaCouture, H.; McCutcheon, C.; Larson, M. J.  (2021) | The impact of exercise intensity on neurophysiological indices of food-related inhibitory control and cognitive control: A randomized crossover event-related potential (ERP) study | NeuroImage | Wrong exposure |
| Barella, L. A.¸ Etnier, J. L.; Chang, Y. K.  (2010) | The immediate and delayed effects of an acute bout of exercise on cognitive performance of healthy older adults. | Journal of Aging and Physical Activity | Wrong population |
| Barr-Anderson, D. J.; AuYoung, M.; Whitt-Glover, M. C.; Glenn, B. A.; Yancey, A. K.  (2011) | Integration of short bouts of physical activity into organizational routine a systematic review of the literature. | American journal of preventive medicine | Wrong publication type |
| Callow, D. D.; Pena, G. S.; Stark, C. E. L.; Smith, J. C.  (2023) | Effects of acute aerobic exercise on mnemonic discrimination performance in older adults. | Journal of the International Neuropsychological Society | Wrong population |
| Carter, S. E.; Draijer, R.; Holder, S. M.; Brown, L.; Thijssen, D. H. J.; Hopkins, N. D.  (2018) | Regular walking breaks prevent the decline in cerebral blood flow associated with prolonged sitting. | Journal of applied physiology | Wrong outcome |
| Carter, S. E.  (2022) | The effects of sitting on cerebrovascular and cognitive function | US : ProQuest Information & Learning (Dissertation) | Wrong publication type |
| Chandrasekaran, B.; Rao, C. R.; Davis, F.; Arumugam, A.  (2021) | SMART STEP - SMARTphone-driven exercise and pedometer-based STEP intervention to promote physical activity among desk-based employees: Study protocol for a three-arm cluster randomized controlled trial. | Work | Wrong publication type |
| Cui, J.; Zou, L.; Herold, F.; Yu, Q.; Jiao, C.; Zhang, Y.; Chi, X.; Müller, N. G.; Perrey, S.; Li, L.; Wang, C.  (2020) | Does cardiorespiratory fitness influence the effect of acute aerobic exercise on executive function? | Frontiers in human neuroscience | Wrong exposure |
| Cuttler, C.; Connolly, C. P.; LaFrance, E. M.; Lowry, T. M.  (2018) | Resist forgetting: Effects of aerobic and resistance exercise on prospective and retrospective memory | Sport, Exercise, and Performance Psychology | Wrong exposure |
| da Silva, W. Q. A.; Fontes, E. B.; Forti, R. M.; Lima, Z. L.; Machado, Dgds; Deslandes, A. C.; Hussey, E.; Ward, N.; Mesquita, R. C.; Okano, A. H.; Elsangedy, H. M.  (2017) | Affect during incremental exercise: The role of inhibitory cognition, autonomic cardiac function, and cerebral oxygenation. | PLoS One | Wrong exposure |
| Drollette, E. S.; Johnson, M. N.; Meadows, C. C.  (2022) | No Change in Inhibitory Control or P3 Following Different High-Intensity Interval Exercise Modalities. | Brain sciences | Wrong exposure |
| Drollette, E. S.; Meadows, C. C.  (2022) | The effects of acute high-intensity interval exercise on the temporal dynamics of working memory and contralateral delay activity. | Psychophysiology | Wrong exposure |
| Dunstan, D. W.; Wheeler, M. J.; Ellis, K. A.; Cerin, E.; Green, D. J.  (2018) | Interacting effects of exercise with breaks in sitting time on cognitive and metabolic function in older adults: Rationale and design of a randomised crossover trial | Mental Health and Physical Activity | Wrong publication type |
| Ehmann, P. J.; Brush, C. J.; Olson, R. L.; Bhatt, S. N.; Banu, A. H.; Alderman, B. L.  (2017) | Active Workstations Do Not Impair Executive Function in Young and Middle-Age Adults. | Medicine and science in sports and exercise | Wrong exposure |
| Ewing, James H.; Gillis, C. A.; Scott, D. G.; Patzig, W. J.  (1982) | Fantasy processes and mild physical activity | Perceptual and Motor Skills | Wrong exposure |
| Frith, E.; Miller, S. E.; Loprinzi, P. D.  (2022) | Effects of Verbal Priming With Acute Exercise on Convergent Creativity. | Psychological reports | Wrong exposure |
| Gejl, A. K.; Bugge, A.; Ernst, M. T.; Tarp, J.; Hillman, C. H.; Have, M.; Froberg, K.; Andersen, L. B.  (2018) | The acute effects of short bouts of exercise on inhibitory control in adolescents | Mental Health and Physical Activity | Wrong population |
| Gilson, N. D.; Hall, C.; Renton, A.; Ng, N.; Hippel, W. v.  (2017) | Do Sitting, Standing, or Treadmill Desks Impact Psychobiological Indicators of Work Productivity? | Journal of Aging and Physical Activity | Wrong exposure |
| Green, D.; Loprinzi, P. D.  (2019) | Experimental Effects of Acute Exercise on Prospective Memory and False Memory. | Psychological reports | Wrong exposure |
| Gu, Q.; Mao, J.; Sun, J.; Teo, W. P.  (2025) | Exercise intensity of virtual reality exergaming modulates the responses to executive function and affective response in sedentary young adults: A randomized, controlled crossover feasibility study. | Physiology & behavior | Wrong comparison |
| Heiland, E. G.; Ekblom, Ö.; Tarassova, O.; Fernström, M.; English, C.; Ekblom, M. M.  (2020) | ABBaH: Activity Breaks for Brain Health. A Protocol for a Randomized Crossover Trial. | Frontiers in human neuroscience | Wrong publication type |
| Hogg, J. A.; Riehm, C. D.; Wilkerson, G. B.; Tudini, F.; Peyer, K. L.; Acocello, S. N.; Carlson, L. M.; Le, T.; Sessions, R.; Diekfuss, J. A.; Myer, G. D.  (2022) | Changes in dual-task cognitive performance elicited by physical exertion vary with motor task. | Frontiers in sports and active living | Wrong comparison |
| Huang, J.; Leng, L.; Hu, M.; Cui, X.; Yan, X.; Liu, Z.; Wang, K.; Wu, J.; He, S.; Deng, W.; Li, P.; Chen, Y.; Gao, D.; Wei, Y.  (2025) | Comparative Effects of Different Exercise Types on Cardiovascular Health and Executive Function in Sedentary Young Individuals. | Medicine and science in sports and exercise | Wrong exposure |
| Jain, P.; Aprajita; J., P.; Jain, A. K.; Babbar, R.  (2014) | Influence of affective changes on behavioral and cognitive performances after acute bout of exhaustive exercise | Journal of Psychophysiology | Wrong exposure |
| Ji, Z.; Feng, T.; Mei, L.; Li, A.; Zhang, C.  (2019) | Influence of acute combined physical and cognitive exercise on cognitive function: an NIRS study. | PeerJ | Wrong population |
| Johnson, L.; Loprinzi, P. D.  (2019) | The effects of acute exercise on episodic memory function among young university students: moderation considerations by biological sex. | Health promotion perspectives | Wrong exposure |
| Kao, S. C.; Baumgartner, N.; Nagy, C.; Fu, H. L.; Yang, C. T.; Wang, C. H.  (2022) | Acute effects of aerobic exercise on conflict suppression, response inhibition, and processing efficiency underlying inhibitory control processes: An ERP and SFT study. | Psychophysiology | Wrong exposure |
| Kao, S. C.; Westfall, D. R.; Soneson, J.; Gurd, B.; Hillman, C. H.  (2017) | Comparison of the acute effects of high-intensity interval training and continuous aerobic walking on inhibitory control. | Psychophysiology | Wrong exposure |
| Kommula, Y.; Callow, D. D.; Purcell, J. J.; Smith, J. C.  (2024) | Acute Exercise Improves Large-Scale Brain Network Segregation in Healthy Older Adults. | Brain Connect | Wrong population |
| Kovacevic, A.; Fenesi, B.; Paolucci, E.; Heisz, J. J.  (2020) | The effects of aerobic exercise intensity on memory in older adults. | Applied Physiology, Nutrition, and Metabolism | Wrong population |
| LaManca, J. J.; Sisto, S. A.; DeLuca, J.; Johnson, S. K.; Lange, G.; Pareja, J.; Cook, S.; Natelson, B. H.  (1998) | Influence of exhaustive treadmill exercise on cognitive functioning in chronic fatigue syndrome. | The American journal of medicine | Wrong population |
| Lamberg, S.; Brakenridge, C. J.; Dunstan, D. W.; Finni, T.; Healy, G. N.; Owen, N.; Pesola, A. J.  (2025) | Electromyography of Sedentary Behavior: Identifying Potential for Cardiometabolic Risk Reduction. | Medicine and science in sports and exercise | Wrong exposure |
| Larson, M. J.; Muir, A. M.; Reid, R. O.; Carbine, K. A.; Marsh, H.; LaCouture, H.; McCutcheon, C.; Bailey, B. W.  (2024) | Does intensity matter? A randomized crossover study of the role of acute exercise intensity on cognitive performance and motor speed and accuracy. | Progress in Brain Research | Wrong exposure |
| Ligeza, T. S.; Maciejczyk, M.; Kałamała, P.; Szygula, Z.; Wyczesany, M.  (2018) | Moderate-intensity exercise boosts the N2 neural inhibition marker: A randomized and counterbalanced ERP study with precisely controlled exercise intensity | Biological Psychology | Wrong exposure |
| Lin, W.; Chen, Q.; Jiang, M.; Tao, J.; Liu, Z.; Zhang, X.; Wu, L.; Xu, S.; Kang, Y.; Zeng, Q.  (2020) | Sitting or Walking? Analyzing the Neural Emotional Indicators of Urban Green Space Behavior with Mobile EEG. | Journal of Urban Health | Wrong outcome |
| Liu, J.; Wei, M.; Li, X.; Ablitip, A.; Zhang, S.; Ding, H.; Zheng, K.; Liu, R.; Ma, X.  (2024) | Substitution of physical activity for sedentary behaviour contributes to executive function improvement among young adults: a longitudinal study. | BMC public health | Wrong exposure |
| Lo Bue-Estes, C.; Willer, B.; Burton, H.; Leddy, J. J.; Wilding, G. E.; Horvath, P. J.  (2008) | Short-term exercise to exhaustion and its effects on cognitive function in young women. | Perceptual and motor skills | Wrong exposure |
| Loprinzi, P. D.; Olafson, D.; Scavuzzo, C.; Lovorn, A.; Mather, M.; Frith, E.; Fujiwara, E.  (2022) | Effects of acute exercise on emotional memory. | Cognition & emotion | Wrong outcome |
| Loprinzi P. D.; Kane, C. J.  (2015) | Exercise and cognitive function: a randomized controlled trial examining acute exercise and free-living physical activity and sedentary effects. | Mayo Clinic proceedings | Wrong exposure |
| Loprinzi, P. D.  (2020) | The effects of acute exercise on episodic memory subtypes: Free, cued, serial recall and recognition | Journal of Cognitive Behavioral Psychotherapy and Research | Wrong exposure |
| Lorås, H.; Haga, M.; Sigmundsson, H.  (2020) | Effect of different exercise modes at high intensity on immediate learning and arousal | International Journal of Sport and Exercise Psychology | Wrong exposure |
| Luteijn, P. J.; van der Wurff, I. S. M.; Singh, A. S.; Savelberg, Hhcm; Groot, R. H. M. de  (2022) | The Acute Effects of Standing on Executive Functioning in Vocational Education and Training Students: The Phit2Learn Study. | Frontiers in psychology | Wrong exposure |
| Lv, Y.; Dong, X.; Sun, T.; Jiang, S.; Gao, Y.; Liang, J.; Hu, S.; Yu, H.; Hou, X.  (2024) | Acute effects of different physical activity on executive function and regulation role of beta oscillation in sedentary youth frontal region. | Scientific reports | Wrong exposure |
| Mainsbridge, C. P.; Cooley, D.; Dawkins, S.; Salas, K. de; Tong, J.; Schmidt, M. W.; Pedersen, S. J.  (2020) | Taking a Stand for Office-Based Workers' Mental Health: The Return of the Microbreak. | Frontiers in public health | Wrong outcome |
| Manci, E.; Theobald, P.; Toth, A.; Campbell, M.; DiFrancisco-Donoghue, J.; Gebel, A.; Müller, N. G.; Gronwald, T.; Herold, F.  (2024) | It's about timing: how density can benefit future research on the optimal dosage of acute physical exercise breaks in esports. | BMJ open sport & exercise medicine | Wrong study design |
| Maylor, B. D.; Hough, J.; Edwardson, C. L.; Zakrzewski-Fruer, J. K.; Bailey, D. P.  (2023) | Stress and Work Performance Responses to a Multicomponent Intervention for Reducing and Breaking up Sitting in Office Workers: A Cluster Randomized Controlled Trial. | Journal of occupational and environmental medicine | Wrong outcome |
| McDonald, K.  (2023) | Cognition, neuroelectric brain function, and salivary biomarkers following exercise | Northeastern University, Massachusetts (Dissertation) | Wrong publication type |
| Medina-Inojosa, J. R.; Gomez Ibarra, M. A.; Medina-Inojosa, B. J.; Supervia, M.; Jenkins, S.; Johnson, L.; Suarez, N. P.; Bonikowske, A.; Somers, V. K.; Lopez-Jimenez, F.  (2024) | Effect of Active Workstations on Neurocognitive Performance and Typing Skills: A Randomized Clinical Trial. | Journal of the American Heart Association | Wrong exposure |
| Morris, T. P.; Fried, P. J.; Macone, J.; Stillman, A.; Gomes‐Osman, J.; Costa‐Miserachs, D.; Tormos Muñoz, J. M.; Santarnecchi, E.; Pascual‐Leone, A.  (2020) | Light aerobic exercise modulates executive function and cortical excitability | European Journal of Neuroscience | Wrong exposure |
| O'Leary, K. C.; Pontifex, M. B.; Scudder, M. R.; Brown, M. L.; Hillman, C. H.  (2011) | The effects of single bouts of aerobic exercise, exergaming, and videogame play on cognitive control. | Clinical neurophysiology: official journal of the International Federation of Clinical Neurophysiology | Wrong exposure |
| Oppezzo, M.; Schwartz, D. L.  (2014) | Give your ideas some legs: the positive effect of walking on creative thinking. | Journal of experimental psychology. Learning, memory, and cognition | Wrong exposure |
| Ozyemisci-Taskiran, O.; Gunendi, Z.; Bolukbasi, N.; Beyazova, M.  (2008) | The effect of a single session submaximal aerobic exercise on premotor fraction of reaction time: an electromyographic study. | Clinical biomechanics (Bristol, Avon) | Wrong exposure |
| Paulus, M.; Kunkel, J.; Schmidt, S. C. E.; Bachert, P.; Wäsche, H.; Neumann, R.; Woll, A.  (2021) | Standing Breaks in Lectures Improve University Students' Self-Perceived Physical, Mental, and Cognitive Condition. | International journal of environmental research and public health | Wrong outcome |
| Pontifex, M. B.; Parks, A. C.; Henning, D. A.; Kamijo, K.  (2015) | Single bouts of exercise selectively sustain attentional processes. | Psychophysiology | Wrong exposure |
| Qi, L.; Wang, G. L.; Yang, Y. L.; Yang, S. Y.; Liu, L. Q.; Zhang, J. W.  (2024) | Positive effects of brisk walking and Tai Chi on cognitive function in older adults: An fNIRS study. | Physiology & behavior | Wrong exposure |
| Salas, C. R.; Minakata, K.; Kelemen, W. L.  (2011) | Walking before study enhances free recall but not judgement-of-learning magnitude | Journal of Cognitive Psychology | Wrong exposure |
| Schmidt-Kassow, M.; Deusser, M.; Thiel, C.; Otterbein, S.; Montag, C.; Reuter, M.; Banzer, W.; Kaiser, J.  (2013) | Physical exercise during encoding improves vocabulary learning in young female adults: a neuroendocrinological study. | PLoS One | Wrong exposure |
| Schwarck, S.; Schmicker, M.; Dordevic, M.; Rehfeld, K.; Müller, N.; Müller, P.  (2019) | Inter-Individual Differences in Cognitive Response to a Single Bout of Physical Exercise-A Randomized Controlled Cross-Over Study. | Journal of clinical medicine | Wrong exposure |
| Shao, X.; He, L.; Liu, Y.; Fu, Y.  (2023) | The effect of acute high-intensity interval training and Tabata training on inhibitory control and cortical activation in young adults. | Frontiers in neuroscience | Wrong exposure |
| Sheahan, P. J.; Diesbourg, T. L.; Fischer, S. L.  (2016) | The effect of rest break schedule on acute low back pain development in pain and non-pain developers during seated work. | Applied ergonomics | Wrong comparison |
| Sibley, B. A.; Etnier, J. L.; Le Masurier, G. C.  (2006) | Effects of an Acute Bout of Exercise on Cognitive Aspects of Stroop Performance | Journal of Sport & Exercise Psychology | Wrong exposure |
| Slusher, A. L.; Patterson, V. T.; Schwartz, C. S.; Acevedo, E. O.  (2018) | Impact of high intensity interval exercise on executive function and brain derived neurotrophic factor in healthy college aged males | Physiology & behavior | Wrong exposure |
| Souza, T. R.; Campos, P. F.; Almeida, M.; Faria, V. M.; Chaves, B. S.; Faria, W. M.; Neves, C. M.; Valentim-Silva, J. R.  (2019) | Exercício progressivo de curtíssima duração possui potente efeito sobre a memória de trabalho, controle inibitório e motricidade fina de adultos jovens sedentários = Progressive exercise of very short duration has a potent effect on working memory, inhibitory control and fine motor skills of sede… | Motricidade | Foreign language |
| Stoner, L.; Willey, Q.; Evans, W. S.; Burnet, K.; Credeur, D. P.; Fryer, S.; Hanson, E. D.  (2019) | Effects of acute prolonged sitting on cerebral perfusion and executive function in young adults: A randomized cross-over trial. | Psychophysiology | Wrong exposure |
| Takahashi, S.; Grove, P. M.  (2023) | Impact of acute open-skill exercise on inhibitory control and brain activation: A functional near-infrared spectroscopy study. | PLoS One | Wrong exposure |
| Thirunavukarasu, E. T.; Reddy, M.; Chandrasekaran, B.; Maiya, A. G.; Rao, C. R.  (2024) | Stair climbing interventions reduce postprandial hyperglycemia but not cognitive functions: findings of a randomized cross-over trial. | Physiology & behavior | Others |
| Thompson, B.; Meynadasy, M.; Hajcak, G.; Brush, C. J.  (2024) | Accelerometer-based and self-reported physical activity and sedentary time and their relationships with the P300 in a Go/No-Go task in older adults. | Brain and cognition | Wrong exposure |
| Tsai, C. L.; Wang, W. L.  (2015) | Exercise-mode-related changes in task-switching performance in the elderly. | Frontiers in behavioral neuroscience | Wrong population |
| Vincent, G. E.; Gupta, C. C.; Sprajcer, M.; Vandelanotte, C.; Duncan, M. J.; Tucker, P.; Lastella, M.; Tuckwell, G. A.; Ferguson, S. A.  (2020) | Are prolonged sitting and sleep restriction a dual curse for the modern workforce? a randomised controlled trial protocol. | BMJ open | Wrong publication type |
| Wanders, L.; Cuijpers, I.; Kessels, R. P. C.; van de Rest, O.; Hopman, M. T. E.; Thijssen, D. H. J.  (2021) | Impact of prolonged sitting and physical activity breaks on cognitive performance, perceivable benefits, and cardiometabolic health in overweight/obese adults: The role of meal composition. | Clinical nutrition (Edinburgh, Scotland) | Wrong population |
| Wang, C. C.; Alderman, B.; Wu, C. H.; Chi, L.; Chen, S. R.; Chu, I. H.; Chang, Y. K.  (2019) | Effects of acute aerobic and resistance exercise on cognitive function and salivary cortisol responses | Journal of Sport and Exercise Psychology | Wrong exposure |
| Waters, A. J.; Burgess, A.; Hughes, D. L.; Jovanovic, J. L.; Miller, E. K.; Li, Y. S.; Basen-Engquist, K. M.  (2012) | Outcome expectancies and expectancy accessibility in exercise behavior | Journal of Applied Social Psychology | Wrong outcome |
| Wilkins, L.  (2024) | Test conditions impact measures of visual, perceptual, and cognitive performance. | Ergonomics | Wrong exposure |
| Wollseiffen, P., Ghadiri, A., Scholz, A., Strüder, H. K., Herpers, R., Peters, T. , Schneider, S.  (2016) | Short Bouts of Intensive Exercise During the Workday Have a Positive Effect on Neuro-cognitive Performance. | Stress and Health: Journal of the International Society for the Investigation of Stress | Wrong exposure |
| Won, J.; Tomoto, T.; Tarumi, T.; Rodrigue, K. M.; Kennedy, K.; Park, D. C.; Zhang, R.  (2025) | Associations of Cardiorespiratory Fitness with Cerebral Cortical Thickness and Gray Matter Volume Across the Adult Lifespan. | Journal of applied physiology | Wrong exposure |
| Won, J.; Wu, S.; Ji, H.; Smith, J. C.; Park, J.  (2017) | Executive Function and the P300 after Treadmill Exercise and Futsal in College Soccer Players. | Sports | Wrong exposure |
| Wu, S.; Ji, H.; Won, J.; Liu, X.; Park, J. J.  (2021) | Effects of Acute Visual Stimulation Exercise on Attention Processes: An ERP Study. | International journal of environmental research and public health | Wrong exposure |
| Zheng, P.; MacDonald, H. V.; Richardson, M. T.; Man, K.; McDonough, I. M.; Aguiar, E. J.  (2024) | Acute Effects of Cadence-Controlled Walking on Cognition and Vascular Function in Physically Inactive Older Adults: A Randomized Crossover Study. | Journal of Aging and Physical Activity | Wrong exposure |
| Zheng, P.  (2023) | The dose-response effect of short-term exercise on cognitive function | University of Alabama Libraries (Dissertation) | Wrong publication type |
| Zukowski, L. A.; Martin, J. M.; Scronce, G.; Lewek, M. D.; Plummer, P.  (2017) | The influence of cognitive load on metabolic cost of transport during overground walking in healthy, young adults. | European journal of applied physiology | Wrong exposure |
